# Melittin-Induced Structural Transformations in DMPG and DMPS Lipid Membranes: A Langmuir Monolayer and AFM Study

**DOI:** 10.3390/molecules29246064

**Published:** 2024-12-23

**Authors:** Joanna Juhaniewicz-Debinska

**Affiliations:** Faculty of Chemistry, Biological and Chemical Research Centre, University of Warsaw, ul. Zwirki i Wigury 101, 02-089 Warsaw, Poland; j.juhaniewicz@uw.edu.pl

**Keywords:** melittin, antimicrobial peptides, ripple phase, model lipid membranes

## Abstract

In this study, we explore the interactions between melittin, a cationic antimicrobial peptide, and model lipid membranes composed of the negatively charged phospholipids 1,2-dimyristoyl-sn-glycero-3-phosphoglycerol (DMPG) and 1,2-dimyristoyl-sn-glycero-3-phosphoserine (DMPS). Using the Langmuir monolayer technique and atomic force microscopy (AFM), we reveal novel insights into these interactions. Our key finding is the observation of the ripple phase in the DMPS bilayer on mica, a phenomenon not previously reported for negatively charged single bilayers. This discovery is significant given the critical role of phosphatidylserine (PS) in cancer biology and the potential of melittin as an anticancer agent. We also highlight the importance of subphase composition, as melittin interacts preferentially with lipids in the liquid-condensed phase; thus, selecting the appropriate subphase composition is crucial because it affects lipid behavior and consequently melittin interactions. Our results show that melittin incorporates into lipid monolayers in both liquid-expanded and liquid-condensed phases, enhancing membrane fluidity and disorder, but is expelled from DMPS in the solid phase. AFM imaging further reveals that melittin induces substantial structural changes in the DMPG membrane and forms the ripple phase in the DMPS bilayers. Despite these alterations, melittin does not cause pore formation or membrane rupture, suggesting strong electrostatic adsorption on the membrane surface that prevents penetration. These findings highlight the differential impacts of melittin on lipid monolayers and bilayers and underscore its potential for interacting with membranes without causing disruption.

## 1. Introduction

Melittin, the main active component in the venom of the European honey bee (Apis mellifera), is a cationic and amphipathic peptide with a net positive charge of +6 at physiological pH, enabling strong electrostatic interactions with negatively charged components of biological membranes [1,2]. This unique property of melittin has made it a subject of extensive research, particularly in understanding its mechanism of action on cell membranes and its potential applications in antimicrobial [2,3], antibiofilm [4,5], hemolytic, and antiviral [6,7] therapies and it is widely researched as an antitumor agent [8,9,10,11]. In aqueous environments, melittin exists in a random coil conformation, but upon encountering cell membranes, lipid vesicles, or surfactant micelles, it adopts a helical structure [12]. This helix formation is independent of the lipid’s physical and chemical properties and is characterized by a hinge at Pro-14, which is essential for melittin activity. The peptide initially binds to lipid membranes as a monomer, a crucial step since tetrameric melittin does not bind effectively to lipid membranes [13]. Once bound, monomers insert into the membrane and aggregate into tetramers upon reaching a critical concentration. Melittin orientation in lipid membranes, either parallel or perpendicular, is influenced by its concentration and the lipid composition of the membrane. Lipid composition profoundly affects melittin action, with zwitterionic membranes being more susceptible to melittin-induced lysis than those with negatively charged lipids [14,15,16,17,18]. Despite melittin’s higher affinity for negatively charged membranes, zwitterionic membranes are more prone to leakage and micellization even at lower melittin concentrations [15,19]. This increased susceptibility is observed across all peptide concentration levels.

Melittin interacts more favorably with negatively charged membranes like those containing phosphatidylserine or phosphatidylglycerol, facilitating parallel orientation that hinders deep penetration into the membrane, thus preventing lysis [20]. In contrast, interactions with zwitterionic membranes rely on hydrophobic forces, allowing deeper penetration and effective membrane disruption [21,22]. Fluorescence studies confirm that melittin penetrates deeper into zwitterionic lipid membranes than into negatively charged ones [23,24,25]. Vibrational spectroscopic studies on phosphatidylglycerol bilayers revealed that both orientations of melittin are present simultaneously, with the parallel orientation being three times more prevalent [26].

Melittin is believed to form toroidal pores in zwitterionic membranes, with the pore size increasing proportionally to the peptide-to-lipid ratio [13,27,28,29]. However, the precise mechanism of melittin interaction with negatively charged membranes remains elusive [30]. Leakage experiments on anionic lipid membranes containing phosphatidylglycerol (PG) lipids have shown no gradual efflux of markers or correlation between melittin concentration and pore size [31]. This suggests that melittin accumulates on the membrane surface, potentially leading to fusion or aggregation and resulting in nonspecific leakage. Ladokhin and White demonstrated that melittin disrupts PG-containing membranes in a detergent-like manner through the carpet mechanism [32]. Conversely, for membranes containing phosphatidylserine (PS), the mode of action of melittin has been reported to depend on both the concentration of melittin and the saturation level of PS lipids [20].

Despite extensive study over the years, melittin continues to be a significant area of interest in biomedical research due to its potent antimicrobial and antitumor properties, which are primarily mediated through its interaction with lipid membranes. For instance, melittin-loaded gold nanocages have shown considerable promise in targeted melanoma therapy by combining cytotoxic and photothermal effects to enhance efficacy [33]. Strategies to reduce melittin cytotoxicity while enhancing its therapeutic efficacy, such as nanomodification and immunoconjugation, have been explored to mitigate its hemolytic side effects [34]. Furthermore, innovative modifications such as tryptophan substitution have enhanced melittin selectivity towards cancer cells while reducing its hemolytic activity [35]. These findings underscore the ongoing importance of studying melittin interactions with lipid membranes, as understanding these interactions is fundamental to optimizing its therapeutic applications and mitigating its side effects.

In this study, we present an integrated characterization of melittin action on negatively charged membranes using Langmuir monolayer techniques and atomic force microscopy. This approach allowed us to examine the interactions between melittin and anionic lipids in different physical states and the melittin-induced structural changes in the organization of supported lipid bilayers. The model lipid membranes were composed of 1,2-dimyristoyl-sn-glycero-3-phosphoglycerol (DMPG) or 1,2-dimyristoyl-sn-glycero-3-phosphoserine (DMPS). We selected these lipids as representatives of anionic lipid components of bacterial and cancer membranes, respectively. Phosphatidylglycerols are common in both gram-positive and gram-negative bacterial membranes [36], while phosphatidylserines are the main anionic lipids in human red blood cells, predominantly located in the inner leaflet of membranes. The externalization of phosphatidylserine to the outer leaflet of the membrane is observed during efferocytosis, infectious disease and cancer [37,38]. However, the choice of DMPG and DMPS as model lipid membranes in our study, while relevant for representing anionic lipid components of bacterial and cancer cell membranes, has inherent limitations. Pure anionic lipid membranes like 100% DMPG or DMPS are not found in nature, which typically contain a mix of lipid types contributing to membrane structure and function [39]. Additionally, the 14:0 acyl chains used in these lipids are uncommon in natural membranes, which usually have mixed acyl chain lengths and degrees of unsaturation [40]. This simplification, while necessary for experimental control, means the findings may not fully replicate natural membrane behavior. Differences in observed interactions suggest factors such as lipid packing density and specific structural interactions play significant roles. However, these lipids provide reproducible π-A isotherms on different subphases and yield excellent results when transferred onto mica to prepare lipid bilayers. Despite these limitations, DMPG and DMPS offer valuable insights into fundamental interactions between melittin and anionic lipids, given their common presence in bacterial membranes and roles in various biological processes. While the exact lipid composition and acyl chain characteristics differ from natural membranes, the chosen lipids still provide a simplified model to study interactions difficult to isolate in complex systems [41].

Notably, our study reveals the formation of a melittin-induced ripple phase in DMPS bilayers on mica, a novel observation that, to the best of our knowledge, has not been reported for negatively charged single bilayers. This finding is particularly significant given the above-mentioned critical role of PS in cancer biology. Additionally, we demonstrate that melittin incorporation into lipid monolayers varies with the lipid phase state. This underscores the necessity of carefully selecting subphase conditions to accurately interpret drug-lipid interactions. These insights advance our understanding of lipid phase behavior and its implications for the interaction of therapeutic peptides and drugs with biological membranes.

## 2. Results

### 2.1. Langmuir Monolayers

The Langmuir trough was employed to study the interactions within the monolayers by measuring surface pressure as a function of molecular area. Surface pressure was monitored using a Wilhelmy plate, which detects changes in surface tension caused by the presence and compression of the monolayer at the air-water interface. The resulting surface pressure-area isotherms provide information about the packing, phase behavior, and interactions of the monolayer components, giving insights into the molecular organization under different conditions.

DMPS and DMPG exhibit similar charge properties in water and buffer solutions, influenced by their pKa values and the ionic environment. In pure water at neutral pH, DMPS typically carries a net negative charge due to the ionization of its serine headgroup. The carboxyl group (-COO^−^) is deprotonated, and the amino group (-NH_3_⁺) is protonated, resulting in a zwitterionic nature, but the overall charge remains negative due to the phosphate group (pKa~1–2) and the deprotonated carboxyl group (pKa~2–3) [42]. Similarly, DMPG in water is also negatively charged, with its phosphate group (pKa~2.9) deprotonated and no additional ionizable groups at physiological pH. When DMPS and DMPG are transferred to a Tris buffer with 100 mM NaCl, the ionic strength increases, which can screen the charges on the lipid headgroups, potentially reducing the electrostatic repulsion between similarly charged groups. However, the fundamental charge of DMPS and DMPG does not change. DMPS will still have a net negative charge due to its phosphate and carboxyl groups, while DMPG will maintain its negative charge from the phosphate group [42]. The presence of Tris buffer and NaCl mainly affects the interaction between lipid molecules and the surrounding ions, but does not fundamentally alter their intrinsic charge states. Despite this ionic screening, the fundamental negative charge of DMPS and DMPG remains unchanged.

**Figure 1:**
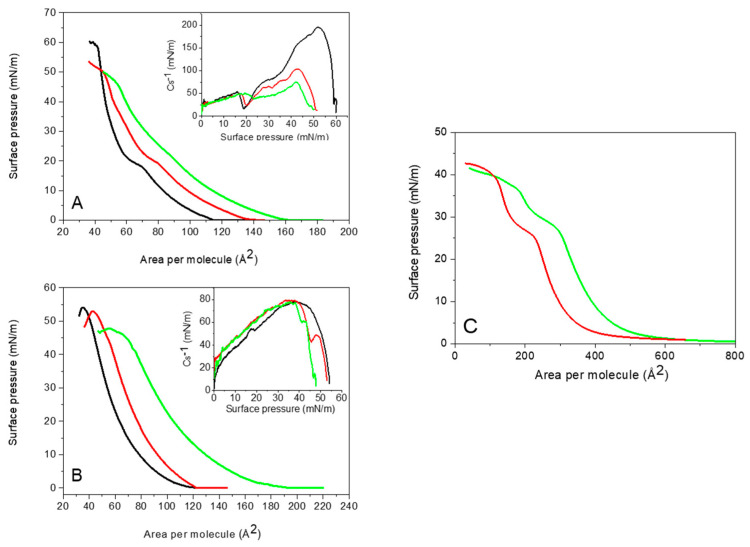
Surface pressure vs. molecular area isotherms of DMPG/MLT mixtures containing 0% (black), 2 mol% (red), and 10 mol% (green) melittin compressed on (**A**) pure water and (**B**) buffer subphase. The inset shows the compression modulus (Cs^−1^) vs. surface pressure plots. (**C**) Surface pressure vs. molecular area isotherms of melittin compressed on pure water (red) and buffer subphase (green).

Melittin is also surface-active and forms monolayers at the air-water interface, even spread from water [43]. The isotherms of melittin compressed on pure water and on a buffer present a similar shape, but they differ in molecular area (Figure 1C). The shift towards higher values of molecular area on buffer results from the presence of ions that preclude the tight packing of melittin molecules due to the electrostatic interactions. Independently of the subphase composition, at high molecular area, monomeric, helical melittin lies parallel to the interface with the hydrophobic faces directed off the water. Once the molecular area is decreased, melittin becomes more densely packed, however, its orientation is still parallel to the interface, and the phase transition-like plateau is observed at approximately 25–30 mN/m. The average molecular area is 350 Å^2^ and 420 Å^2^ for water and the buffer, respectively, which is in agreement with crystallographic data reported by Terwilliger and coworkers [44]. Further compression leads to a change in orientation from flat lying to tilted and then perpendicular to the subphase. The minimum area required for a melittin molecule is approximately 120 Å^2^ and 190 Å^2^ for water and the buffer, respectively, suggesting that the final orientation of melittin is indeed close to perpendicular with respect to the interface. Assuming that the diameter of α-helix is 12 Å, its projection area on the surface would be 113 Å^2^. Melittin is known to adopt a conformation of bent helical rod. Therefore, the expected area will be slightly higher. Further compression results in a small increase in the surface pressure because melittin molecules are forced down into the subphase.

At room temperature and on the water subphase, DMPG undergoes a phase transition from the liquid-expanded (LE) phase to the liquid-condensed (LC) phase, observable as a plateau region at approximately 18–21 mN/m [45]. Figure 1A shows typical π-A isotherms of DMPG and its mixtures with melittin compressed on a water subphase. Increasing concentrations of melittin shift the π-A isotherms towards higher molecular area values, resulting in an increase in the mean molecular area (Figure 1A and Table 1). Melittin presence affects the LE-LC phase transition, making it less pronounced and eventually disappearing completely at 10 mol% melittin. These changes are also evident in the compression modulus (Cs^−1^) plots, where the phase transition appears as a minimum, and the maximum value corresponds to the monolayer physical state. In the presence of melittin, DMPG becomes more fluid, altering the LE-LC phase transition (inset in Figure 1A and Table 1). Additionally, melittin significantly decreases the collapse pressure (π_coll_). To elucidate these effects, the excess area of mixing (A^exc^) and excess Gibbs energy of mixing (ΔG^exc^) were calculated using the following formulas:

$$\;A^{\mathrm{exc}}=A_{12}-\left(x_{1}A_{1}+x_{2}A_{2}\right)$$

where A_12_ is the mean area per molecule in the mixed monolayer, A_1_ and A_2_ are the molecular areas of single components, and x_1_ and x_2_ are the mole fractions of the components. Further analysis on the interaction between components is based on the excess Gibbs energy of mixing (ΔG^exc^), which can be easily evaluated from the following equation:

$$\;{\Delta G}^{\mathrm{exc}}=N_{A}\int_{0}^{\pi }A^{\mathrm{exc}}d\pi$$

where N_A_ is the Avogadro’s constant equal to 6.023 × 10^23^ mol^−1^.

The excess area of mixing A^exc^ quantifies deviations from ideal mixing behavior in a monolayer system. If the components mix ideally, the area per molecule in the mixed monolayer would be a weighted average of the areas of the individual components. Positive values of A^exc^ indicate repulsive interactions between the components, where the molecules occupy more space than expected, while negative values suggest attractive interactions, leading to a more compact packing. The excess Gibbs energy of mixing ΔG^exc^ provides a thermodynamic perspective on these interactions by integrating A^exc^ over the range of surface pressures. This quantity reflects the work required to compress the monolayer and highlights whether the mixing interactions are energetically favorable (negative ΔG^exc^) or unfavorable (positive ΔG^exc^). Together, A^exc^ and ΔG^exc^ offer insights into the structural and thermodynamic properties of the mixed monolayer, revealing the strength and nature of molecular interactions.

**Figure 2:**
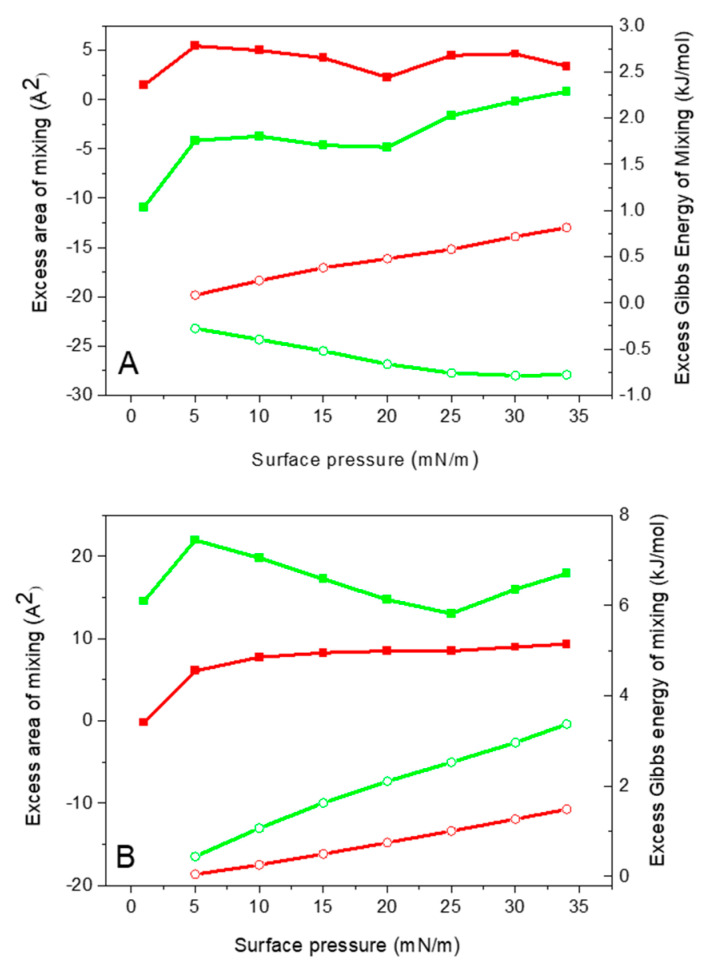
Excess area (squares) and excess Gibbs energy of mixing (circles) vs. surface pressures of DMPG/MLT mixed monolayers containing 2 mol% (red) and 10 mol% (green) melittin compressed on (**A**) pure water and (**B**) buffer subphase.

At 2 mol% melittin, the values of both A^exc^ and ΔG^exc^ are positive, indicating repulsive interactions within the monolayer (Figure 2A). These repulsions likely arise from interactions between the hydrophilic side of melittin and the hydrophobic acyl chains, which dominate over melittin-lipid electrostatic interactions. Conversely, at 10 mol% melittin, electrostatic attraction between melittin and DMPG becomes predominant, leading to condensation and stabilization of the monolayer, as indicated by negative values of A^exc^ and ΔG^exc^. However, the values calculated for both mixtures are relatively small, suggesting that both repulsive and attractive interactions are rather weak.

The presence of the buffer solution in the subphase significantly influences DMPG monolayers, leading to an increase in mean molecular area and a decrease in collapse pressure (Figure 1B and Table 1). Additionally, DMPG compressed on a buffer does not exhibit a phase transition at room temperature. This complex behavior of DMPG is associated with its phase transition properties: at low ionic strength, DMPG shows a broad phase transition region between 18 and 35 °C, where an intermediate phase exists, and a fully fluid state is achieved above 35 °C [46]. In contrast, in solutions containing more than 100 mM NaCl, DMPG undergoes the LE-LC transition at 23 °C, resembling the behavior of zwitterionic 1,2-dimyristoyl-sn-glycero-3-phosphocholine (DMPC) [46,47]. Therefore, in the presence of a buffer containing 150 mM NaCl at 22 °C, the phase transition is not observed on the π-A isotherm, as reflected in the compression modulus plot. The maximum Cs^−1^ value is approximately 78 mN/m, indicating that the DMPG monolayer exists in the liquid-expanded phase.

Increasing melittin concentration significantly increases the mean molecular area (Table 1). The collapse pressure decreases with higher melittin content in the monolayer, indicating lower stability of mixed monolayers compared to pure DMPG monolayers (Figure 1B). Although the system is above the collapse pressure of pure melittin, the isotherms of the mixed monolayers do not overlap with the isotherm of pure DMPG. This indicates that melittin is incorporated into the monolayer and contributes to the observed behavior. The presence of melittin disrupts the cohesive interactions within the DMPG monolayer, leading to a reduction in the overall stability of the mixed monolayer compared to the pure DMPG monolayer. The observed decrease in collapse pressure with increasing melittin content reflects the altered packing and stability of the system as a whole, rather than the collapse of individual components. Analysis of the excess functions of mixing revealed that interactions within the DMPG/MLT mixed monolayers on the buffer subphase are predominantly repulsive, as indicated by the positive values of A^exc^ and ΔG^exc^ (Figure 2B). These results suggest that, while lipid-peptide mixing is thermodynamically unfavorable, the repulsive interactions are relatively weak. The small deviations from ideal mixing suggest that melittin mixes with DMPG without causing major structural changes to the monolayer. At the same time, the presence of melittin weakens the “average” interactions between the lipids, which reduces the overall stability of the monolayer. This balance between weak repulsive interactions and maintained structural integrity shows how melittin interacts with DMPG in a way that affects the properties of the monolayer without completely disrupting it.

The analysis of the excess area of mixing confirms that melittin interactions with lipid membranes strongly depend on the lipid phase behavior. Without a buffer, DMPG transitions from the liquid-expanded (LE) phase to the LC phase. At 2% mol melittin, the excess area of mixing is similar on both water and buffer subphases, indicating weakly repulsive interactions due to melittin’s probable perpendicular orientation to the lipid monolayer, slightly disrupting lipid packing. However, at 10% mol melittin, the melittin reorients parallel to the lipid headgroups, leading to stronger electrostatic attractions with the negatively charged DMPG headgroups. This results in more preferential interactions, with a smaller excess area reflecting dominant attractive forces. In the presence of a buffer, the DMPG monolayer remains in the LE phase, similar to zwitterionic DMPC. The excess area of mixing is positive and increases with increasing melittin content, indicating repulsive interactions. The ions in the buffer screen the negative charge on DMPG, diminishing electrostatic interactions between DMPG headgroups and melittin, allowing melittin to occupy more space within the monolayer. This suggests melittin is oriented perpendicularly and increasingly incorporates into the membrane. These results indicate that melittin interactions are more favorable with DMPG in the liquid-condensed phase in comparison to the liquid-expanded phase. These conclusions align with other studies on mixed monolayers of melittin with various phosphocholine lipids, which also found that melittin-lipid interactions are more favorable with lipids in the liquid-condensed phase than in the liquid-expanded phase [48].

**Figure 3:**
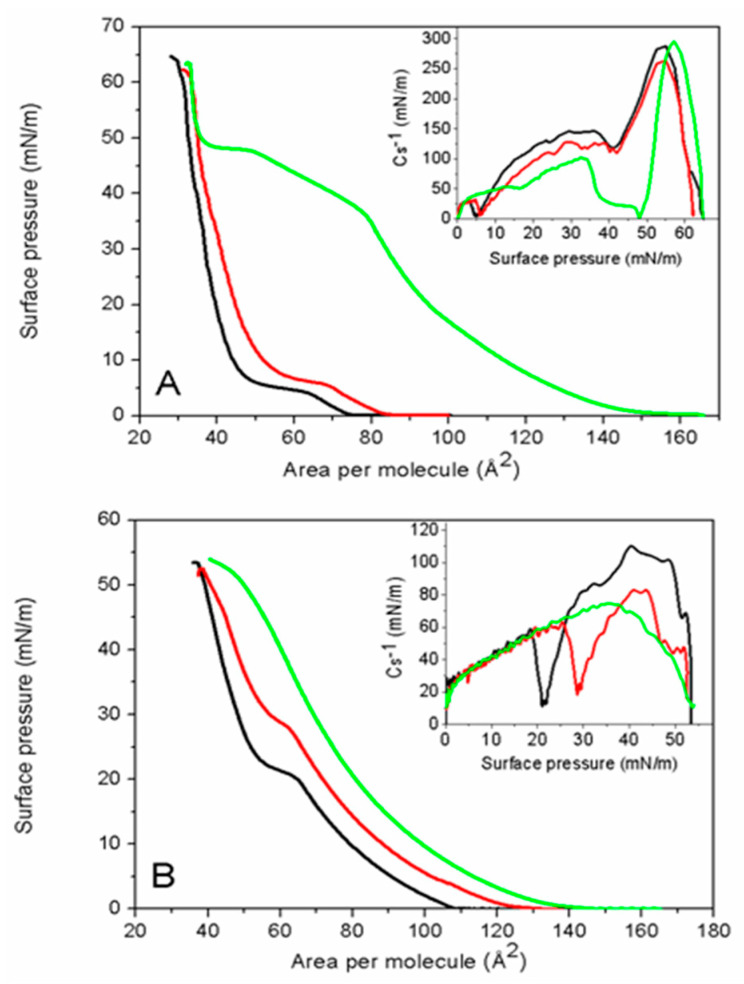
Surface pressure vs. molecular area isotherms of DMPS/MLT mixtures containing 0% (black), 2 mol% (red), and 10 mol% (green) melittin compressed on (**A**) pure water and (**B**) buffer subphase. The insets show the compression modulus (Cs^−1^) vs. surface pressure plots.

Figure 3A shows the π-A isotherms of DMPS and DMPS/MLT mixtures compressed on water. DMPS undergoes an LE-LC phase transition, visible as a plateau region approximately of 5–6 mN/m [49]. However, the compression modulus vs. surface pressure plot (inset in Figure 3A, black curve) reveals two minima at 5 mN/m and 40 mN/m, indicating phase transitions. The first minimum corresponds to the LE-LC phase transition, while the second is likely associated with the formation of a solid phase. The maximum Cs^−1^ value (287 mN/m) supports this interpretation.

The influence of melittin on the properties of DMPS differs significantly from its effect on DMPG. At 2 mol% melittin, only a slight increase in the mean molecular area is observed, along with minor changes in the collapse pressure and molecular area. The LE-LC phase transition is minimally affected by the presence of melittin, as confirmed by the compression modulus plots. The minimum corresponding to the LE-LC phase transition shifts slightly towards higher surface pressures, while the position of the second minimum remains unchanged. Additionally, there is only a minor decrease in the maximum compression modulus value, indicating that DMPS remains largely in the solid phase and is relatively insusceptible to 2 mol% melittin. This behavior may be due to the tightly packed DMPS molecules, which make interactions with melittin unfavorable, forcing the peptide molecules into the subphase. This is also evident from the π-A isotherms, where the difference in molecular area between pure DMPS monolayers and those containing 2 mol% melittin decreases with increasing surface pressure, indicating peptide expulsion into the subphase.

The surface properties of mixed monolayers containing 10 mol% melittin are even more complex. In this case, melittin causes a significant shift in the π-A isotherm towards larger molecular areas. The π-A isotherm and compression modulus plot show three distinct regions. The first region, between 0 and 35 mN/m, corresponds to the mixed monolayer of lipids and peptides. The substantial shift of the π-A isotherm towards higher molecular areas and the decrease in compression modulus values indicate that melittin inserts into the DMPS film, making it more fluid. The second region, observed between 35 and 50 mN/m, is a plateau associated with the expulsion of melittin molecules into the subphase. The third region, between 50 and 63 mN/m, represents an almost pure DMPS monolayer, overlapping with the π-A isotherm of the 2 mol% melittin mixture, indicating a small amount of melittin remaining at the interface. This conclusion is supported by the increase in compression modulus values, indicating a return to the lipid solid phase. The π-A isotherm shape, with two collapses, clearly indicates the immiscibility of the components within the monolayer [50]. Moreover, it confirms the repulsive and unfavorable interactions between melittin and DMPS in the solid phase.

**Figure 4:**
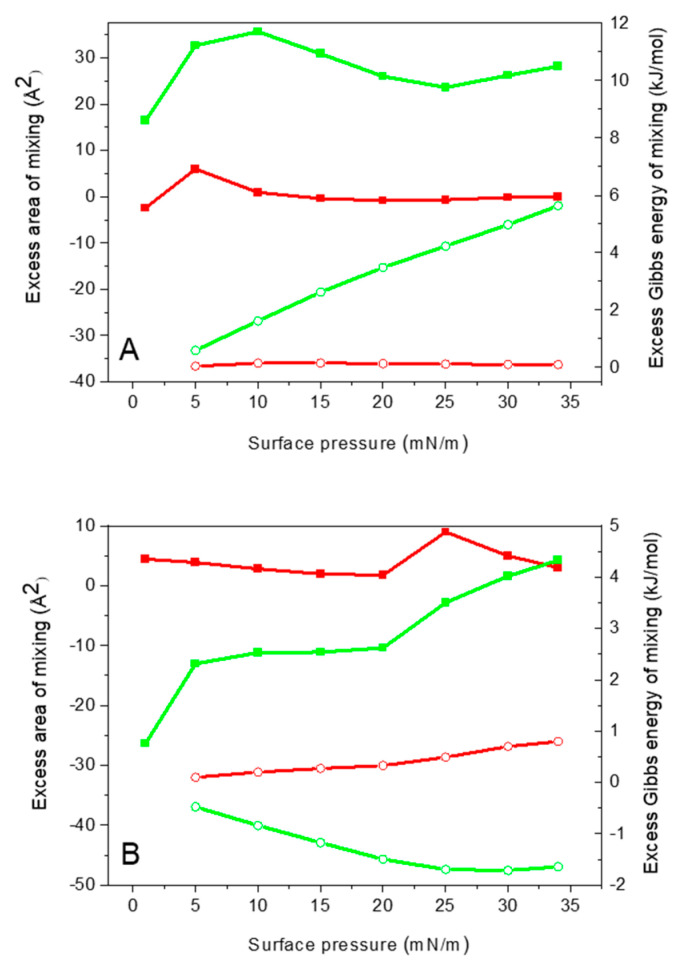
Excess area (squares) and excess Gibbs energy of mixing (circles) vs. surface pressures of DMPS/MLT mixed monolayers containing 2 mol% (red) and 10 mol% (green) melittin compressed on (**A**) pure water and (**B**) buffer subphase.

The analysis of A^exc^ and ΔG^exc^ values confirms the conclusions drawn from π-A isotherms and compression modulus plots (Figure 4A). For the monolayer containing 2 mol% melittin, both parameters are close to zero, indicating either immiscibility or ideal miscibility of the components. However, since the physical state of DMPS remains unchanged, immiscibility of DMPS and melittin is more probable. In contrast, at 10 mol% melittin, strong repulsive interactions are observed between the monolayer components, as indicated by the positive values of A^exc^ and ΔG^exc^. These values suggest that mixing between DMPS and melittin is energetically unfavorable. Combined with the findings from the π-A isotherms and compression modulus, which reveal distinct regions corresponding to changes in monolayer composition, this indicates that phase separation may occur under these conditions. Since the π-A isotherm indicates that at high surface pressure melittin is expelled from the monolayer into the subphase, it is observed that phase separation is likely a result of 3D segregation rather than in-plane separation. These results are consistent with results obtained for mixtures of DMPC/CHOL (7:3) with melittin, which also form solid-phase monolayers at room temperature but undergo fluidization in the presence of melittin [51]. This significant difference in melittin influence on DMPS and DMPC/CHOL monolayers suggests that negatively charged DMPS is more resistant to melittin action.

On a water subphase (Figure 4A), the excess area of mixing is positive and increases with higher melittin content, indicating repulsive interactions. This is counterintuitive because, without charge screening, one would expect the electrostatic attraction between negatively charged DMPS and positively charged melittin to dominate, resulting in a negative excess area. However, the positive excess area suggests that DMPS molecules are densely packed, creating a tightly ordered structure. When melittin is introduced, it disrupts this packing due to its amphipathic nature and likely perpendicular orientation, interacting with the lipid tails and polar headgroups. This orientation causes steric hindrance and hydration forces that increase the area per molecule, outweighing electrostatic attractions and leading to repulsive interactions. Thus, the repulsive interactions, driven by steric effects, hydration forces, and the structural incompatibility between densely packed DMPS and melittin, dominate, resulting in a positive excess area of mixing.

The presence of buffer solution in the subphase shifts the DMPS LE-LC phase transition to approximately 20 mN/m (Figure 3B). This shift is caused by sodium ions in the buffer that screen the charge on anionic monolayers. Monovalent cations decrease the temperature of the lipid phase transition and fluidize the membrane at a given temperature [52]. The maximum compression modulus of DMPS is approximately 120 mN/m, indicating that DMPS exists in the liquid-condensed phase (inset in Figure 3B and Table 1). Additionally, the mean molecular area increases due to the presence of counterions, and the collapse pressure decreases (Table 1), confirming the lower stability of DMPS monolayers on buffer compared to pure water. Increasing concentrations of melittin cause the π-A isotherms to shift towards larger molecular areas (Figure 3B). Melittin presence also affects the LE-LC phase transition, which shifts to significantly higher surface pressures and eventually disappears with increasing melittin concentration. These changes are also evident in the compression modulus plots (inset in Figure 3B). The decrease in maximum compression modulus indicates that the membrane fluidity increases with higher melittin concentrations.

Calculated values of A^exc^ and ΔG^exc^ confirm the different behavior of DMPS/MLT monolayers on a buffer subphase compared to water (Figure 4B). At 2 mol% melittin, both parameters are positive, indicating repulsive interactions and immiscibility of the monolayer components. In contrast, at 10 mol% melittin, attractive interactions predominate, resulting in negative values of excess area and excess Gibbs energy of mixing. This suggests that melittin interactions with negatively charged lipids in mixed monolayers are most favorable when the lipids are in the liquid-condensed phase. This leads to increased miscibility of monolayer components and the formation of a homogeneous monolayer. The liquid-condensed phase facilitates electrostatic interactions between the polar fragments of the peptide and the lipids, which predominate over repulsions between hydrophobic and hydrophilic regions. In the solid phase, lipids push the peptide into the subphase to maintain monolayer stability.

Interestingly, at 2% mol melittin, the excess area values are similar on both water and buffer subphases for both DMPS and DMPG, suggesting that at low melittin content, the system behavior is not significantly influenced by the subphase composition. However, as melittin content increases to 10% mol, the trends become the opposite. On the buffer subphase, the excess area of mixing decreases, indicating more attractive interactions. This reversal can be attributed to the presence of ions in the buffer solution, which screen the electrostatic charges on DMPS, thereby reducing the electrostatic repulsion between lipid molecules. In this environment, melittin likely adopts a more parallel orientation to the monolayer. This orientation promotes better packing and reduces steric hindrance as melittin content increases. Moreover, for DMPS, the higher phase transition temperature (Tm) and the presence of both negatively charged phosphate and carboxyl groups lead to stronger headgroup-headgroup interactions, resulting in a more rigid and tightly packed structure that resists melittin insertion. This rigid organization causes the peptide to be expelled from the monolayer at higher surface pressures, reflecting unfavorable interactions between melittin and the solid-phase DMPS. In contrast, DMPG, with a Tm more than 10 °C lower than that of DMPS, forms a more fluid monolayer with weaker headgroup interactions, facilitating the incorporation of melittin. Additionally, DMPG has a single negatively charged group, which reduces repulsive forces between headgroups and allows for tighter lipid-peptide packing, even when electrostatic screening by buffer ions is present. These findings underscore the critical role of lipid phase state, headgroup interactions, and the interplay between electrostatic and nonelectrostatic forces in determining the miscibility and stability of peptide-lipid mixed monolayers.

To evaluate melittin’s ability to incorporate into existing lipid membranes, we performed stability experiments at two different surface pressures: 15 and 35 mN/m. These values were chosen to verify the role of the lipid physical state, with the pressure of biological membranes estimated to be approximately 35 mN/m [53]. At 15 mN/m, both DMPG and DMPS exist in the liquid-expanded phase, while at 35 mN/m, DMPG remains in the liquid-expanded phase and DMPS forms a liquid-condensed phase monolayer. Stability experiments were conducted on pure lipid monolayers and after injecting melittin for at least 8 h (Figure S1, supporting information). For both initial surface pressures, an increase in surface pressure was observed after melittin injection. However, the magnitude of this increase is directly affected by the lipid physical state. At 15 mN/m, melittin incorporation into both DMPG and DMPS was facilitated, leading to an identical increase in surface pressure of 24 mN/m. This similar behavior is expected, as both lipids are negatively charged and form liquid-expanded phase monolayers. When lipids were precompressed to 35 mN/m, melittin injection led to a significantly lower increase in surface pressure: 10 mN/m for DMPG and 8 mN/m for DMPS. These values indicate that the tight packing of lipid molecules within the monolayer reduces melittin’s ability to penetrate lipid membranes. The more condensed organization of molecules within the DMPS monolayer further inhibits melittin incorporation, resulting in a lower increase in surface pressure. The maximum insertion pressure (MIP) of melittin was estimated to be 40 and 42 mN/m for DMPS and DMPG, respectively (Figure S2, supporting information), indicating similar susceptibility of both lipids to melittin action. These values are significantly lower than the MIP determined for the zwitterionic DMPC membrane, proving that negatively charged membranes are more resistant to melittin action [51,54]. However, all presented MIP values are above the lateral pressure of biological membranes, indicating that melittin disrupts both bacterial and eukaryotic cell membranes nonselectively.

### 2.2. Atomic Force Microscopy

In order to monitor melittin-induced structural changes in the lipid membranes, MAC mode atomic force microscopy (AFM) was employed. Lipid bilayers were transferred on mica, which provides a significantly smoother surface than gold substrates. Since DMPG and DMPS are negatively charged, prior to the lipid deposition, the mica was modified with APTES to create a positive charge at the surface of the substrate and consequently facilitate the transfer of lipid bilayers from the Langmuir trough.

**Figure 5:**
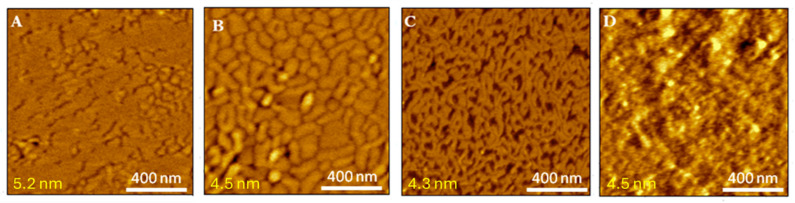
MAC mode topography images of the DMPG bilayer after (**A**) 0 min, (**B**) 1 h, (**C**) 3 h, and (**D**) 12 h of exposure to 10 μM melittin. The thickness of the membrane is shown in the left lower corner (for details see supporting information).

Figure 5A shows that the mica surface is covered with a lipid membrane, indicating the successful transfer of DMPG layers. The thickness of the DMPG bilayer before exposure to melittin is estimated to be 5.2 ± 0.1 nm. To quantitatively describe melittin-induced structural changes in lipid membranes, the RMS roughness (the standard deviation of the surface heights from an average height on the imaged surface; see supporting information for the equation) was determined. The DMPG bilayer surface is smooth with an RMS roughness of 0.13 nm, though several cracks with a depth of 0.4 nm are observed (Figure 5A). Therefore, the membrane thickness in the area of such defects is smaller, which indicates the local coexistence of liquid-ordered and liquid-disordered phases, similar to DMPC bilayers near the main transition temperature [55].

Exposure of the DMPG membrane to a 10 μM melittin solution induces significant structural changes. Ten minutes after melittin addition, the number of cracks increases, and a “brain-like” structure is observed with an RMS roughness of 0.21 nm. The height difference from cross-sectional analysis ranges from 0.4 to 0.5 nm. This structure remains stable for approximately 2 h (Figure 5B). Further exposure leads to a labyrinth-like structure (Figure 5C) with lipid stripes averaging 20 nm in width and a height difference of approximately 1 nm (RMS roughness of 0.31 nm). After 12 h, the membrane becomes highly irregular and disordered, reflected in an RMS roughness of 0.70 nm (Figure 5D). However, even after this time, no rupture of the DMPG membrane is observed.

The morphological changes are likely due to the fluidizing effect of melittin on the DMPG bilayer. Melittin strongly adsorbs onto the DMPG surface due to electrostatic interactions between its cationic residues and the negatively charged lipid headgroups. Melittin may preferentially adsorb at the borders between LC and LE phases, propagating the membrane fluidization and forming the “brain-like” structure. The increasing fraction of the LE phase leads to the formation of a labyrinth-like corrugated structure, eventually resulting in a complete transition of the DMPG membrane to the fluid phase with melted acyl chains, observed as a disordered structure in Figure 5D. However, even in the fluid phase, melittin does not induce pore formation, and the membrane remains stable 12 h after the addition of 10 μM melittin. This underscores the importance of electrostatic interactions in melittin action on lipid membranes.

**Figure 6:**
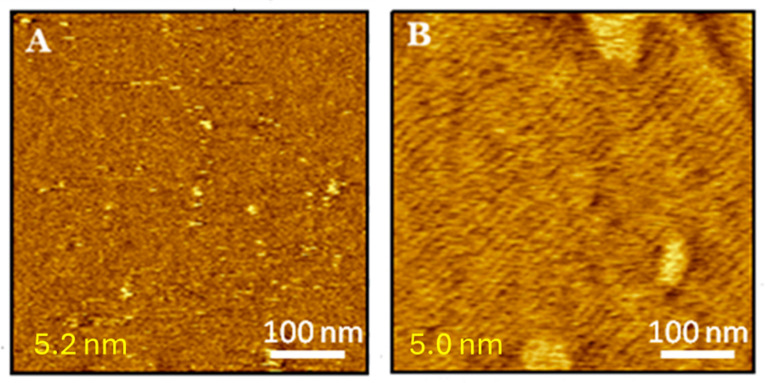
MAC mode topography images of the DMPS bilayer after (**A**) 0 min, (**B**) 15 min of exposure to 10 μM melittin. The thickness of the membrane is shown in the left lower corner (for details see supporting information).

The transfer of DMPS from the air-water interface results in the formation of a well-packed, homogeneous, and smooth bilayer with an average thickness of 5.2 ± 0.2 nm (Figure 6A). DMPS forms more condensed layers than DMPG. The topography image of the DMPS bilayer in Figure 6A shows that the mica surface is covered with a very compact bilayer, likely in a liquid-condensed/solid phase, indicating that deposition on a solid support does not substantially affect the physical state of the DMPS bilayer. The RMS roughness for the pure DMPS membrane is 0.15 nm.

Adding 10 μM melittin induces structural perturbations in the DMPS bilayer. Fifteen minutes after melittin addition (Figure 6B), undulations indicative of a ripple phase (P_β’_) are observed, remaining stable and essentially unchanged for several hours. The ripples are regular and uniformly distributed across the imaged surface, with a periodicity of 12 ± 3 nm and a peak-to-valley height difference of 0.6 ± 0.2 nm based on cross-sectional analysis. While these observations strongly suggest the formation of a ripple phase, there could be doubts regarding the nature of the lamellar structures seen in the AFM images. One alternative interpretation might involve elongated micelles. However, geometric considerations help clarify this ambiguity. For elongated micelles, the width would correspond to the doubled length of the DMPS lipid molecules—approximately 5 nm, which matches the expected micelle diameter. In contrast, the periodicity of the observed structures is significantly larger, approximately 12 nm. Of course, one can argue that these are double-layered micelles, and then the width of the lamellae could be approximately 12 nm. Nevertheless, the thickness of the film should be analogous (i.e., ~12 nm), while it is approximately 5 nm. These geometric arguments strongly support the conclusion that the observed features are not micelles, but rather a ripple phase. To the best of our knowledge, this is the first report of ripple phase formation in a phosphatidylserine bilayer. An additional argument that this is indeed P_β’_ is the phase images obtained simultaneously with the surface topography presented in the supporting information. Because phase imaging in AFM detects variations in material properties like stiffness and viscoelasticity, it can reveal differences between ripple-phase regions and other areas in a lipid bilayer. Since the observed features correspond to periodic contrasts in the phase image—matching the known patterns of the ripple phase—this confirms that the features are indeed due to the ripple phase.

Lipids are known to exist in various phases depending on temperature, composition, and hydration levels [56,57]. A periodic ripple phase (P_β’_) is observed as an intermediate phase between the gel (L_β’_) and liquid-crystalline (L_α_) phases within a narrow temperature range between the pretransition and main phase transition temperatures [58]. The formation of the ripple phase has been reported for saturated phosphatidylcholines [59,60] and their mixtures [61], membranes containing unsaturated lipids [62], phosphatidylglycerol [63], and sphingomyelin [64]. For many years, it has been suggested that the ripple phase does not occur in symmetric, solid-supported, single bilayers on mica substrates due to substrate-lipid interactions significantly influencing lipid bilayer behavior [55,64]. AFM has been used to observe the ripple phase in asymmetric lipid bilayers, the top bilayers of supported double bilayers, and floating bilayer lipid membranes (fBLM) [65,66]. However, the ripple phase has been observed in a symmetric single DMPC bilayer immobilized on gold [67]. The results presented in this article demonstrate the formation of the ripple phase in a single, symmetrical DMPS bilayer transferred onto a mica substrate. The DMPS bilayer is immobilized on APTES-modified mica, which could affect the formation of the ripple phase by potentially altering the interaction between the substrate and the lipid bilayer. However, no ripple phase was observed before the addition of melittin. Ripples formed just a few minutes after melittin addition, without being preceded by substantial structural changes in the membrane. The behavior of the DMPS membrane can be explained as follows:

Firstly, the DMPS bilayer does not remain in direct contact with the solid support, as the mica was modified with APTES molecules before transferring the lipid bilayer. Secondly, melittin strongly adsorbs onto the DMPS surface due to electrostatic interactions. The high concentration of melittin on the DMPS surface may lead to the reorientation of individual melittin molecules that incorporate into the DMPS bilayer. However, this penetration does not disrupt the membrane integrity, which remains highly impermeable to buffer ions but may slightly reduce its solidity. Additionally, since the DMPS membrane is in a highly condensed physical state, melittin adsorption may induce local curvature of the DMPS surface, resulting in ripple formation. It is also possible that the adsorbed melittin generates packing frustrations in the membrane, leading to the formation of ripples to relieve these perturbations.

Moreover, the condensed or gel lipid phase appears crucial for ripple phase formation. Although the DMPG membrane is also negatively charged and separated from the mica surface by the APTES layer, the ripple phase does not occur due to the higher fluidity of the DMPG bilayer compared with the DMPS membrane. Thus, it can be concluded that melittin may induce ripple phase formation in solid-supported single bilayers, but this is possible only when the membrane is strongly hydrated and exists in a condensed or gel phase.

It is important to emphasize the different time courses of melittin interactions with DMPG and DMPS membranes. The first visible changes in the morphology of the DMPG membrane occur 15–30 min after exposure to melittin, similar to DMPS. However, the DMPS membrane maintains its ripple phase structure regardless of exposure time, reaching a stationary state relatively quickly. In contrast, the DMPG membrane continues to change until it undergoes degradation and partial dispersion. This slower progression to a steady state may result from the looser packing density of lipid molecules in DMPG compared to DMPS. The denser packing in the DMPS membrane hinders melittin penetration, making it more difficult to achieve the critical lipid-to-peptide ratio required for membrane dispersion. Conversely, the lower density in DMPG facilitates more peptide accumulation, resulting in a longer penetration time. The differences between DMPS and DMPG membranes may also be due to the varying stability of the lipid-melittin complexes and the distinct ways melittin incorporates into these lipid structures. A similar effect has been observed for DMPS and DMPG membranes deposited on gold [54].

## 3. Materials and Methods

1,2-dimyristoyl-sn-glycero-3-phosphoglycerol (DMPG) and 1,2-dimyristoyl-sn-glycero-3-phosphoserine (DMPS) were purchased from Avanti Polar Lipids Inc., Alabaster, AL, USA. Melittin, tris(hydroxymethyl)aminomethane (TRIS), sodium chloride, ethylenediaminetetraacetic acid (EDTA), and (3-aminopropyl)triethoxysilane (APTES) were obtained from Sigma Aldrich, Darmstadt, Germany. All reagents and compounds were used without further purification. Distilled water for all experiments was purified using a Milli-Q system, achieving a final resistivity of 18.2 MΩ/cm.

Monolayer experiments at the air-water interface were performed using a KSV LB Trough 5000 (KSV Ltd., Espoo, Finland) and a NIMA Trough (NIMA Technology Ltd., Coventry, UK), both equipped with two movable Teflon barriers and a surface pressure sensor. Before each experiment, troughs and barriers were cleaned with a chloroform-methanol mixture and rinsed with Milli-Q water. Monolayers were formed on either pure water or a subphase containing a buffer solution of 20 mM TRIS, 150 mM NaCl, and 5 mM EDTA, adjusted to pH 7.5 ± 0.1 with HCl. Monolayer compression was performed at a barrier speed of 10 mm/min at a constant temperature of 22 ± 1 °C. DMPG, DMPS, and melittin were dissolved in a methanol-chloroform mixture (1:3 *v*/*v* for lipids and 1:2 *v*/*v* for melittin). After cleaning the subphase and spreading the lipid or lipid/peptide solution at the interface, the solvent was allowed to evaporate for 20 min. After that, compression surface pressure-mean molecular area (MMA) isotherms were recorded. In the case of lipid-melittin mixtures, the MMA represents the averaged calculated area for the mixed film. Each π-A isotherm was repeated at least three times to confirm its reproducibility. Preparing the mixture of compounds in chloroform before spreading it at the air-water interface is a common procedure. However, to exclude the potential peptide-lipid interactions resulting from preparing mixed solutions in chloroform, the reference experiment was performed in which melittin was spread after the spreading of lipids. The results from both approaches were consistent within experimental errors.

For experiments involving melittin injection into the subphase, melittin stock solution was prepared by dissolving it in pure water. The incorporation of melittin into preformed DMPG and DMPS model membranes was investigated at two surface pressures: 15 mN/m and 35 mN/m, corresponding to different physical states of lipids. DMPG and DMPS monolayers were formed on the buffer solution subphase. After compressing the lipid monolayers to the selected surface pressure, the barriers were stopped to maintain a constant area, melittin was injected into the subphase to a final concentration of 0.3 μM, and changes in surface pressure over time were recorded.

To determine the maximum insertion pressure (MIP) of melittin, lipids were spread at the air-buffer interface to reach initial surface pressures (π_i_) of 5 mN/m, 10 mN/m, 15 mN/m, and 20 mN/m. Melittin was injected into the subphase to a final concentration of 0.3 μM, and the increase in surface pressure was monitored until it reached a steady-state value. The difference between equilibrium and initial pressure (Δπ) was calculated, and a plot of Δπ vs. π_i_ was constructed. Extrapolating this plot to the x-axis provided the MIP value.

For atomic force microscopy (AFM) experiments, lipid bilayers were transferred onto mica discs (20 mm diameter, Ted Pella, Inc., Redding, CA, USA), which were cleaved with adhesive tape before deposition. Due to the negative charges on mica surfaces, transferring negatively charged DMPG and DMPS was inefficient. To address this, mica surfaces were modified with APTES to generate positive charges. The procedure involved filling a desiccator with argon, washing mica discs in chloroform, drying them, and placing them in the desiccator with vessels containing 30 μL of APTES and 10 μL of triethylamine. The reaction was left for 2 h, after which the vessels were removed, the desiccator was purged with argon, and the mica discs were left overnight to cure. This resulted in surface amine group modifications, partially protonated under experimental conditions.

Lipid bilayers were transferred from the air-water interface onto solid supports at a pressure of 35 mN/m. The first lipid layer was deposited by vertically withdrawing the substrate at a speed of 5 mm/min to achieve a transfer ratio of 1.0 ± 0.1. The substrates were dried for approximately 1.5 h, and the second lipid layer was transferred at a surface pressure of 35 mN/m using the horizontal touch technique (Langmuir–Schaefer method). For each lipid, six samples have been prepared for AFM studies.

AFM images were taken with an Agilent 5500 AFM (Agilent Technologies, Santa Clara, CA, USA). All experiments were conducted at room temperature in a buffer solution (20 mM TRIS, 150 mM NaCl, and 5 mM EDTA, pH 7.5 ± 0.1). Prior to each experiment, the liquid cell was cleaned in piranha solution (concentrated H_2_SO_4_/30% H_2_O_2_, 3:1 *v*/*v*) for at least 2 h and thoroughly rinsed with water. Images were taken in MAC-Mode (Magnetic Alternating Current mode) using MAC cantilevers, type VII (Agilent Technologies), with a nominal spring constant of 0.14 N/m. The bilayer-modified mica was mounted in the liquid cell, and the lipid membrane was imaged multiple times to ensure no disruption occurred due to imaging. Subsequently, melittin solution was injected into the liquid cell to a final concentration of 10 μM, and structural changes in membrane topography were imaged over time.

## 4. Conclusions

This study demonstrates the profound impact of melittin on anionic lipid monolayers, specifically DMPG and DMPS, highlighting the critical role of lipid phase state and electrostatic interactions. Melittin significantly increases the fluidity of DMPG monolayers, suppressing the liquid-expanded (LE) to liquid-condensed (LC) phase transition and reducing collapse pressure, with these effects amplified in a buffer subphase. Conversely, DMPS monolayers exhibit resistance to melittin incorporation, maintaining their integrity and solid phase, with higher melittin concentrations causing phase separation and repulsive interactions. Thermodynamic analyses reveal that melittin interactions with DMPG transition from repulsive to attractive at higher concentrations while remaining consistently repulsive with DMPS. Stability experiments at biologically relevant pressures indicate that tightly packed lipid molecules hinder melittin incorporation, as confirmed by AFM. AFM studies reveal substantial structural changes in DMPG bilayers upon melittin exposure, transitioning from smooth surfaces to highly irregular, disordered structures after prolonged exposure, demonstrating melittin’s strong fluidizing effect without causing membrane rupture. Conversely, melittin induces a periodic ripple phase in DMPS bilayers, indicating local curvature and packing frustrations, while the membrane retains its overall integrity and highly impermeable nature. These findings offer new insights into the potential of melittin as an anticancer agent. By disrupting lipid packing and inducing structural reorganization in lipid membranes, melittin could destabilize the membrane integrity of target cells, leading to functional impairments and promoting cell death. Additionally, the observed ripple phase and packing frustrations in DMPS bilayers may serve as a model for understanding how melittin interacts with specific lipid domains in cellular membranes, paving the way for future studies to optimize its therapeutic applications. This detailed understanding of melittin modulation of membrane stability and fluidity underscores the importance of lipid phase state and electrostatic interactions, providing valuable insights into peptide-lipid interactions.

## Figures and Tables

**Table 1:** Characteristic parameters of pure lipid and lipid/melittin mixed monolayers formed on different subphases.

Monolayer (Subphase)	A_0_ (Å^2^)	A_coll_ (Å^2^)	π_coll_ (mN/m)	π_LE-LC_ (mN/m)	Max Cs^−1^ (mN/m)
DMPG (water)	56.5 ± 0.2	46.9 ± 0.5	59.3 ± 0.3	18.6 ± 0.5	198 ± 3
2% MLT	70.0 ± 0.3	48.6 ± 0.3	51.4 ± 0.8	19.6 ± 0.5	108 ± 2
10% MLT	101.5 ± 0.6	54.2 ± 0.7	48.8 ± 0.5	------	75 ± 1
DMPG (buffer)	76.0 ± 0.6	38.0 ± 0.4	54.2 ± 0.5	------	78 ± 1
2% MLT	96.5 ± 0.7	47.2 ± 0.7	52.9 ± 0.7	------	78 ± 1
10% MLT	130.4 ± 0.5	69.4 ± 0.8	47.3 ± 0.6	------	78 ± 2
DMPS (water)	42.4 ± 0.7	31.2 ± 0.4	65.2 ± 0.5	5.4 ± 0.2	288 ± 3
2% MLT	45.9 ± 0.4	34.3 ± 0.3	63.1 ± 0.7	7.3 ± 0.3	269 ± 3
10% MLT	45.0 ± 0.5	36.4 ± 0.5	63.9 ± 0.6	------	290 ± 3
DMPS (buffer)	62.0 ± 0.6	36.6 ± 0.2	53.4 ± 0.6	20.4 ± 0.2	113 ± 3
2% MLT	71.5 ± 0.7	38.6 ± 0.4	52.4 ± 0.3	28.3 ± 0.4	84 ± 2
10% MLT	96.0 ± 0.5	42.6 ± 0.3	53.5 ± 0.4	------	76 ± 2

## Data Availability

The data presented in this study are available upon request from the corresponding author.

## Supplementary Materials

The following supporting information can be downloaded at: https://www.mdpi.com/article/10.3390/molecules29246064/s1, Equations used for the thickness of lipid membranes. Figure S1: Stability experiments; Figure S2: Maximum insertion plots; Figure S3: AFM images and cross-sectional profiles; Table S1: Changes in membrane thickness upon interaction with melittin.; Figure S4: AFM images of DMPS bilayer after exposure to 10 µM melittin illustrating topography (A,C) and corresponding phase images (B,D).
