# Supplementary material for: Melittin-Induced Structural Transformations in DMPG and DMPS Lipid Membranes: A Langmuir Monolayer and AFM Study
Source: Molecules. 2024 Dec 23;29(24):6064. doi: 10.3390/molecules29246064 (PMC11677270; doi:10.3390/molecules29246064)
Supplement: Supplementary file 1 [file molecules-29-06064-s001.zip › molecules-3344334-supplementary.pdf]

Supporting Materials

Melittin-Induced Structural Transformations in DMPG and DMPS Lipid Membranes: A Langmuir Monolayer and AFM Study

Joanna Juhaniewicz-Debinska

Faculty of Chemistry, Biological and Chemical Research Centre, University of Warsaw, ul. Zwirki i Wigury 101, 02-089 Warsaw, Poland; j.juhaniewicz@uw.edu.pl

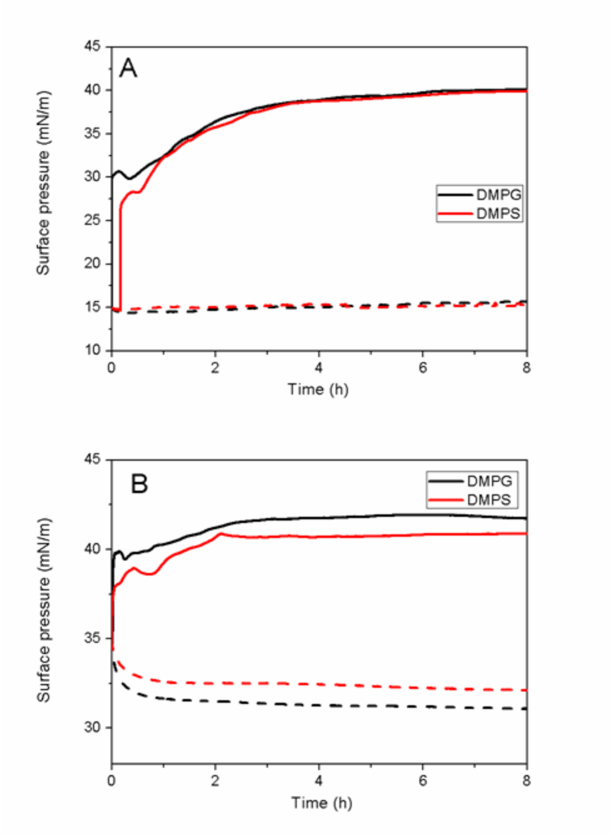

Figure S1. Changes in surface pressure in time of lipid monolayer formed on pure buffer (dashed) and after injection of melittin (solid). The monolayers were compressed to 15 mN/m (A) and 35 mN/m (B).

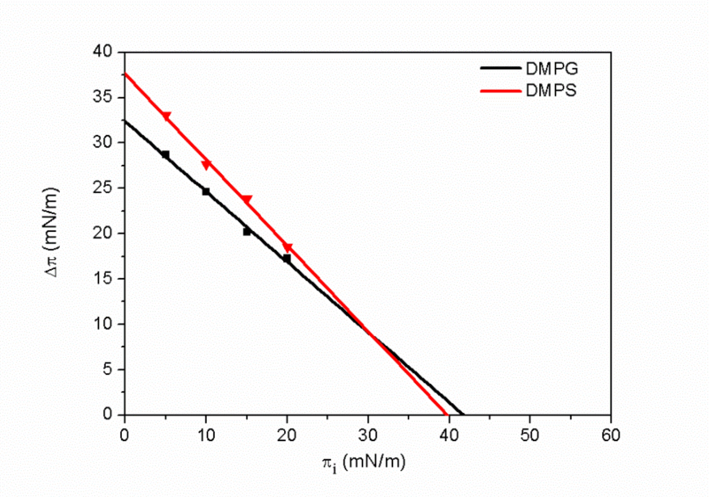

Figure S2. Changes in surface pressure vs. initial surface pressure. The extrapolation of plots allows determining MIP value.

RMS roughness for each topography image was determined according to the equation:

$$RMS = \sqrt{\frac{1}{NM}\sum_{x=0}^{N-1}\sum_{y=0}^{M-1} z_{x,y}^2}$$

where N is the number of points per scan line, M is the number of scan lines, $z_{x,y}$ is the amplitude at point (x,y).

The thickness of the lipid membranes before and after exposure to melittin was obtained from cross-sectional profiles taken along membrane defects.

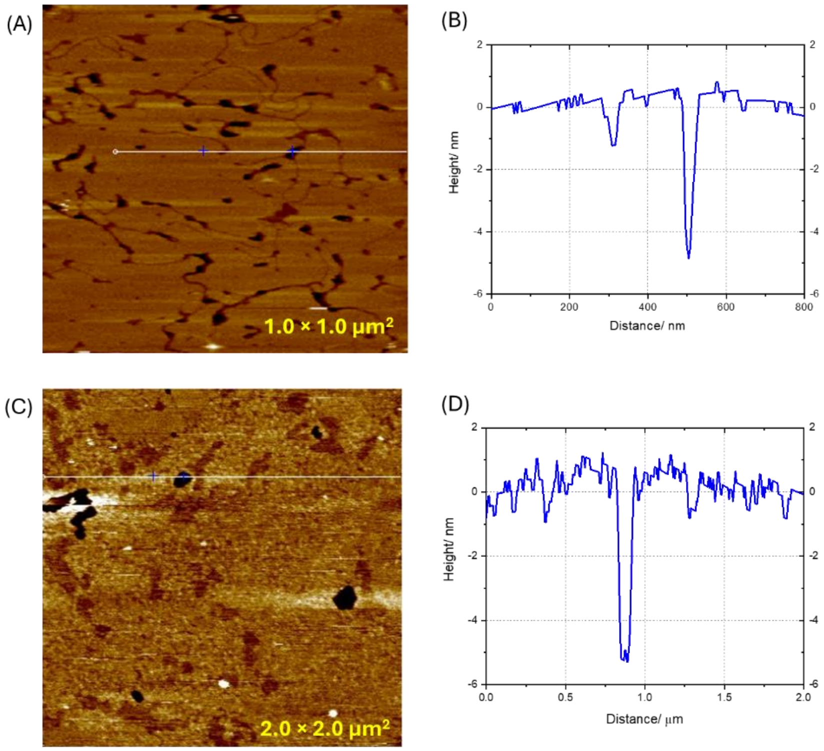

Figure S3. AFM images of DMPG (A) and DMPS (C) membranes and corresponding cross sectional profiles taken along the defects for DMPG bilayer (B) and DMPS bilayer (D) .

Table S1. Thickness of the membranes exposed to 10 μM melittin determined from cross sectional profiles taken along the defects in the film.

Time of exposure	Thickness of DMPG membrane (nm)	Thickness of DMPS membrane (nm)
0 minutes	5.2 ± 0.1	5.2 ± 0.2
15 minutes	5.2 ± 0.3	5.0 ± 0.6
60 minutes	4.5 ± 0.9	no further changes
180 minutes	4.3 ± 0.7	no further changes
12 hours	4.5 ± 1.2	no further changes

By using phase imaging in conjunction with topography, AFM provides a robust means of identifying and confirming the ripple phase in lipid bilayers. The periodic nature of the ripple phase should manifest as both topographic undulations and corresponding periodic contrasts in the phase image. The phase image provides information about the material's mechanical and adhesive properties. This makes phase imaging a valuable tool for verifying whether observed features in a lipid bilayer correspond to the ripple phase. In the ripple phase, the periodic features are typically associated with differences in material properties (e.g., stiffness, adhesion, or viscoelasticity) between the crests and troughs of the ripples. The phase image highlights these differences, as the AFM tip experiences varying energy dissipation when interacting with regions of differing mechanical properties. If the observed ripples in the topographic image truly represent the ripple phase, the phase image should exhibit periodic contrast corresponding to the same spatial frequency. The AFM images recorded for DMPS membrane upon exposure to melittin are shown in Figure S4. Phase imaging (Figure S4B and S4D) confirm the periodicity and shape of the ripples.

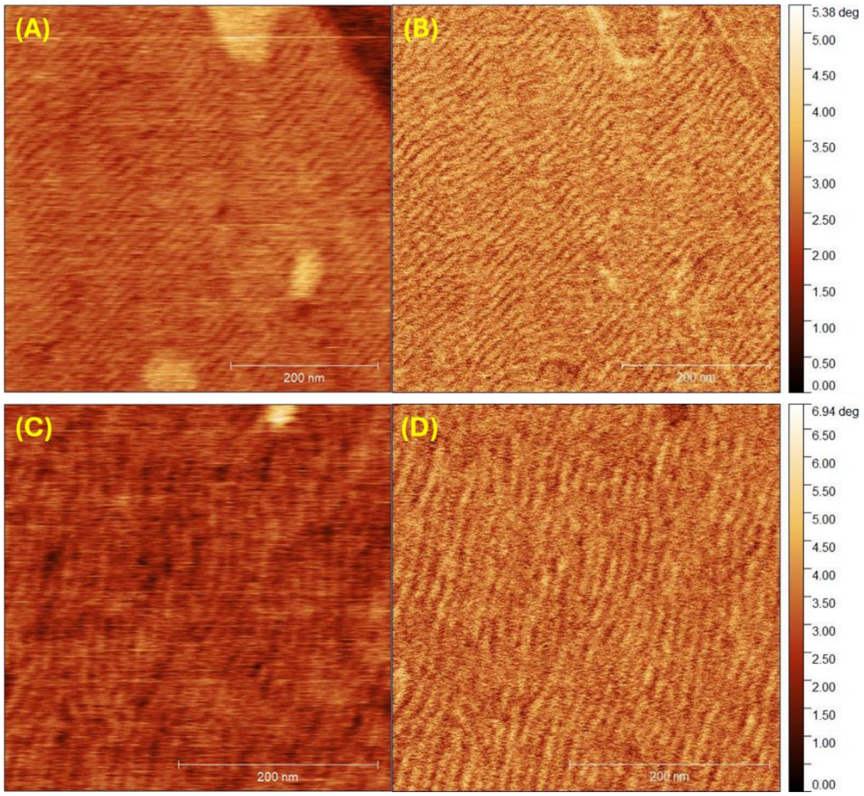

Figure S4. AFM images of DMPS bilayer after exposure to 10 μM melittin illustrating topography (A, C) and corresponding phase images (B, D).
